# Osteoidosis leads to altered differentiation and function of osteoclasts

**DOI:** 10.1111/jcmm.15227

**Published:** 2020-04-13

**Authors:** Lisanne Grünherz, Carina Prein, Thomas Winkler, Manuela Kirsch, Ursula Hopfner, Thomas Streichert, Hauke Clausen‐Schaumann, Jozef Zustin, Kristin Kirchhof, Michael M. Morlock, Hans‐Günter Machens, Arndt Friedrich Schilling

**Affiliations:** ^1^ Experimental Plastic Surgery Clinic for Plastic and Hand Surgery Technische Universität München Munich Germany; ^2^ Center for Applied Tissue Engineering and Regenerative Medicine (CANTER) Munich Germany; ^3^ Department of Applied Sciences and Mechatronics Munich University of Applied Sciences Munich Germany; ^4^ Institute of Biomechanics Technische Universität Hamburg‐Harburg Hamburg Germany; ^5^ Department of Clinical Chemistry University Hospital of Cologne Cologne Germany; ^6^ Gerhard Domagk Institute of Pathology University Medical Center Muenster Muenster Germany; ^7^ Biomet Deutschland GmbH Berlin Germany; ^8^ Department of Trauma Surgery, Orthopedic Surgery and Plastic Surgery University Medical Center Göttingen Göttingen Germany

**Keywords:** mechanotransduction, osteoclast, osteomalacia, RGD peptide, vitamin D

## Abstract

In patients with osteomalacia, a defect in bone mineralization leads to changed characteristics of the bone surface. Considering that the properties of the surrounding matrix influence function and differentiation of cells, we aimed to investigate the effect of osteoidosis on differentiation and function of osteoclasts. Based on osteomalacic bone biopsies, a model for osteoidosis in vitro (OIV) was established. Peripheral blood mononuclear cells were differentiated to osteoclasts on mineralized surfaces (MS) as internal control and on OIV. We observed a significantly reduced number of osteoclasts and surface resorption on OIV. Atomic force microscopy revealed a significant effect of the altered degree of mineralization on surface mechanics and an unmasking of collagen fibres on the surface. Indeed, coating of MS with RGD peptides mimicked the resorption phenotype observed in OIV, suggesting that the altered differentiation of osteoclasts on OIV might be associated with an interaction of the cells with amino acid sequences of unmasked extracellular matrix proteins containing RGD sequences. Transcriptome analysis uncovered a strong significant up‐regulation of transmembrane glycoprotein TROP2 in osteoclastic cultures on OIV. TROP2 expression on OIV was also confirmed on the protein level and found on the bone surface of patients with osteomalacia. Taken together, our results show a direct influence of the mineralization state of the extracellular matrix surface on differentiation and function of osteoclasts on this surface which may be important for the pathophysiology of osteomalacia and other bone disorders with changed ratio of osteoid to bone.

## 1 INTRODUCTION

Normal bone function requires bone remodelling that is a lifelong and complex process involving bone formation and bone resorption. During this, the osteoclast plays a pivotal role due to its unique ability of bone resorption. Osteoclasts belong to the mononuclear phagocyte system and originate from HSCs through differentiation of CD14+ monocytes.^1^, ^2^ During differentiation, osteoclastogenesis requires two essential factors: macrophage colony‐stimulating factor (M‐CSF) and receptor activation of NF‐kB ligand (RANKL). M‐CSF mainly promotes proliferation and survival of osteoclast precursors. RANKL is known to function as the primary factor driving differentiation of osteoclast precursors by controlling gene expression by activating its receptor RANK. Secondary, osteoclast function highly depends on bone‐cell interaction and its cytoskeleton organization. Bone‐cell interaction is mediated by integrin avß3 which recognizes the RGD sequence that is present in various bone matrix proteins. Interaction of integrin avß3 with the bone matrix induces cytoskeleton organization that leads to polarization of the osteoclast and establishment of the typical resorptive compartment.^3^ Any imbalance in the regulation of bone remodelling can result in a metabolic bone disease like osteoporosis, osteopetrosis, osteosclerosis, pycnodysostosis or osteomalacia.^4^, ^5^, ^6^, ^7^, ^8^, ^9^

Vitamin D deficiency, in particular, is an increasingly important global health problem across all ages. It leads to changes in the metabolism of calcium that eventually result in decreased mineralization of bone.^10^ Based on histological findings, accumulation of unmineralized bone tissue (osteoid) is referred to as osteoidosis. According to Parfitt, the resulting condition with a ratio of osteoid volume to bone volume >10% is called osteomalacia.^11^

In a cross‐sectional study of a normal German population, osteomalacia occurred in more than 25%, independent of the subjects age.^12^ The degree of mineralization largely determines the stiffness of a tissue. Thus, osteoidosis leads to a decreased elastic modulus (Young's modulus) of the affected bone surface.^13^ Therefore, it can be supposed that the matrix mechanics for the bone cells which are attached to the bone surface are modified by this disorder. For a variety of cells, it has already been shown that the matrix properties can influence cellular functions, including cell proliferation, locomotion, adhesion, spreading, morphology, striation and differentiation.^14^, ^15^, ^16^, ^17^, ^18^, ^19^, ^20^ For haematopoietic stem cells (HSCs) in particular, Lee‐Thedieck et al have shown that their migration and adhesion behaviour are dependent on the elastic modulus of the substrate. Similar effects are reported by Holst et al who described an altered proliferation behaviour of HSCs caused by an altered Young's modulus of the surrounding substrate.^21^, ^22^

If the differentiation of HSCs can be influenced by the substrate's elasticity, we have been suggested that the differentiation and function of osteoclasts might also be influenced by changes of the bone surface. Therefore, we investigated the effect of matrix mineralization on the differentiation and function of osteoclasts.

## 2 MATERIALS AND METHODS

### 2.1 Morphometric analysis of osteomalacia

After fixation in buffered formalin, the tissues were embedded undecalcified in methyl‐methacrylate, cut into sections of 5 µm thickness using a k‐microtome (Jung) and stained with Goldner trichrome staining. The retrospective analysis of patient's records was performed in compliance with the Hamburg Hospital Law, Germany (HmbKHG, Version: 17th of April, 1991; Second chapter: Patients data protection, Paragraph 12).

### 2.2 In vitro model of mineralized tissue and OIV

Dentin is widely used as a model for mineralized tissue. It was provided by German customs in accordance with the international laws for the protection of species. Discs of 10.0 × 10.0 × 0.7 mm were prepared by using a diamond saw (PSI Grünewald GmbH & Co. KG). Physiologically mineralized dentin discs (Mineralized surface, MS) were served as control. To generate an unmineralized matrix and imitate an osteoidosis (OIV), the surface of dentin discs was demineralized by immersion in one molar hydrochloric acid (Carl Roth GmbH & Co. KG) for 1 minute. After demineralization, the dentin was washed thoroughly with distilled water and stored at room temperature (RT) until its use in cell culture.

### 2.3 Preparation of RGD peptide‐coated dentin

Dentin discs were prepared as described above. The cyclic RGD peptide c(RGDfK) (RGD, Novabiochem) was solubilized in MilliQ water (Merck MilliPore) to give a concentration of 100 µmol/L. Each disc was covered with 500 µL RGD solution and incubated for 10 minutes under ambient conditions. Finally, discs were cleaned with 100% isopropanol for 5 minutes and dried for 3 hours inside the laminar flow hood.

### 2.4 Atomic force microscopy

Atomic force microscopy (AFM) measurements were carried out as previously described.^23^ Measurements were carried out on a NanoWizard AFM (JPK Instruments) combined with an Axiovert 200 inverted optical microscope (Carl Zeiss AG). For contact mode imaging and elasticity measurements, sharp pyramidal silicon nitride cantilevers (MLCT Microcantilever, Bruker) with 0.02 N/m nominal spring constant and tip half‐opening angle of 17.5° were used. The force constant of each cantilever was determined individually using the thermal noise method. Images of the osteoidosis model and physiologically mineralized dentin discs were acquired in air with a scan rate of 1 Hz and a resolution of 512 × 512 pixels. All elasticity measurements were carried out for three different discs in PBS (pH 7.4). 25 × 25 indentation points over an image section of 10 × 10 µm were chosen, and force vs distance curves were recorded at 1 Hz. After converting force vs distance curves to force vs indentation curves, the Hertz model, modified for a pyramidal tip geometry, was used to extract the elastic modulus (Young's modulus) at each indentation point.^24^

$$F=\frac{2}{\pi }*\tan\alpha *\frac{E}{\left(1-\nu^{2}\right)}*\delta^{2}$$

For conversion of force vs distance to force vs indentation curves, as well as Young's modulus extraction, the JPK Data Processing Software version 4.2.20 (JPK Instruments AG) was used. Finally, Igor Pro Software version 6.0.2.4 (WaveMetrics, Inc) was used to visualize the frequency distribution of measured Young's moduli in a histogram. The centre of the distribution was determined by fitting a Gaussian distribution to the data. For further analysis of the surface, the D‐period of the collagen network was determined using cross‐sectional AFM images and the Gwyddion Software 2.26.

### 2.5 Cell culture of human osteoclasts

Peripheral blood mononuclear cells (PBMCs) were isolated from buffy coats of anonymous, healthy, voluntary blood donors, giving informed consent. Buffy coat is a waste product of the process of blood component manufacturing and was used in accordance with the ethical standards of the institutional research committee and with the 1964 Helsinki declaration and its later amendments. As described previously, density gradient centrifugation with Ficoll‐Paque was used to separate the mononuclear osteoclast precursor cells from other formed elements in the buffy coat. To dispose the culture of lymphocytes, the cells were purified for adherence. Cells were then cultivated at a density of 2 × 10^6^ cells/mL on prepared dentin discs in alpha MEM containing 1% l‐glutamine, 1% penicillin/streptomycin/amphotericin (PAA Laboratories GmbH), 10% foetal calf serum (Gibco), 20 ng/mL M‐CSF and 40 ng/mL RANKL (PeproTech GmbH) for up to 28 days.^25^

### 2.6 TRAP staining and evaluation of osteoclast numbers

After 28 days of cultivation, cells on dentin discs were fixed with 37% buffered formaldehyde and incubated with TRAP staining solution (Sigma Aldrich). Then, cells were analysed with the Eclipse TS 100 inverted optical microscope combined with a digital camera and the NIS‐Elements BR software (Nikon GmbH). To determine the number of osteoclasts, cells positive for TRAP and with three or more nuclei were counted. The experiment was repeated for three times, each time including six individual replicates.

### 2.7 Infinite focus microscopy

The three‐dimensional resorption activity of osteoclasts on MS, OIV and RGD‐coated dentin was quantified by infinite focus microscopy (IFM) as described by us previously.^26^ Briefly, cells were detached from the surface after 28 days of cultivation. For three‐dimensional quantification of the resorbed volume, three‐dimensional surface data sets were generated with voxel sizes of 800 nm × 800 nm × 100 nm using IFM (InfiniteFocus Alicona). To minimize the effect of surface roughness on measured resorption, a roughness exclusion plane was defined as the mean of all depth values of the topographic surface of the untreated material minus two times standard deviation. Only pits which were deeper than this roughness exclusion plane were included in the analysis. Pseudocolour visualization as well as computation of resorbed volume, resorbed area and depth was performed with the built‐in software in combination the freeware image analysis software (UTHSCSA Image Tool V 3.0). Measurements were repeated with three different samples for each surface topography.

### 2.8 mRNA microarray analysis

Microarray analysis was performed after 28 days of cultivation using the Human Genome U133 Plus 2.0 Arrays (Affymetrix). Procedures for cDNA synthesis, labelling and hybridization were carried out according to the manufacturer's protocol (Affymetrix). In brief, 50 ng of total RNA was used for first‐strand cDNA synthesis with an HPLC‐purified T7‐(dT)24 primer. Synthesis of biotin‐labelled cRNA and clean up was carried out using the IVT Express Kit (Affymetrix). For hybridization, 15 µg of fragmented cRNA was incubated with the chip in 200 µL of hybridization solution in Hybridization Oven 640 (Affymetrix) at 45°C for 16 hours. GeneChips were then washed and stained using the Affymetrix Fluidics Station 450 according to the GeneChip Expression Analysis Technical Manual (Rev. 2). Microarrays were scanned with the Affymetrix GeneChip Scanner 7G, and the signals were processed using GCOS (v.1.4; Affymetrix).

### 2.9 Quantitative realtime PCR analysis

At day 28, RNA isolation was carried out using the High Pure RNA Cell Isolation Kit (Roche Diagnostics GmbH) following the manufacturer's instructions. RNA was then transcribed to cDNA in the iQ5 qPCR cycler by the use of 5× iScript RT Supermix (Bio‐Rad Laboratories). For qPCR analysis, 2 ng of resulting cDNA of each probe was used. The performed PCR protocol was as follows: one step of 2 minutes at 98°C, 40 primer‐specific cycles of 10 seconds at 95°C and 30 seconds at 60°C, a melt curve was generated with 80 cycles of 6 seconds and a starting temperature of 65°C with 0.5°C increments.

Primer: TROP2 forward 5′‐TGG CCT ACC CGA GGA GAA GAG GA‐3′, reverse 3′‐CGT TCA GGC AGC TGA AAC AGG CT‐5′. The amplification of the housekeeping gene 28S rRNA, forward 5′‐CGG TTT CGC GAG CGC GTT G‐3′ and reverse 3′‐ATA GCC GCA ACC GGA CCC TGG‐5′, served as reference. In order to quantify the relative change of gene expression, the ΔΔCt‐method was used. Thus ΔCt was calculated by normalizing the Ct (cycle of threshold) value to the 28S rRNA Ct value. Afterwards, the ratio of ΔCt (=2^ΔCt^) was determined to express the gene expression relative to 28S rRNA. qPCR analysis was repeated for three times. Each experiment included twelve replicates per group (mineralized dentin vs OIV) that were pooled prior to RNA isolation.

### 2.10 TROP2 immunohistochemistry staining

Cells on MS and OIV were fixed with 3.7% formaldehyde. Afterwards, cells were incubated for 1 hour at RT with PBS containing 10% BSA, followed by incubation of the primary antibody (Cat.: ab79976, Abcam GmbH) in 1% BSA in PBS (1:200) overnight. The next day, samples were incubated with the secondary antibody (Cat.: A11019, Life Technologies GmbH) in 1% BSA in PBS (1:1000). Finally, cells were counterstained using DAPI (DAPI, Cat.: P26931, Life Technologies GmbH) and pictures were taken with the fluorescence microscope Eclipse TE 2000‐S (Nikon GmbH). Osteomalacia bone biopsies were subjected to the same procedure.

### 2.11 Statistical analysis

All experiments were repeated for three times to allow statistical analysis, except for the mRNA microarray, which was performed once with a pooled sample. Statistical analysis and the generation of graphics were performed using Sigma Plot software. For normally distributed data, one‐way ANOVA testing was performed, followed by Tukey post hoc testing, if there were more than two groups. For non‐normally distributed data, Mann‐Whitney rank sum test was performed. The probability of a type I error was set to 5% (*α* = .05). Error bars represent SD.

## 3 RESULTS

### 3.1 Model for osteoidosis in vitro

**FIGURE 1:**
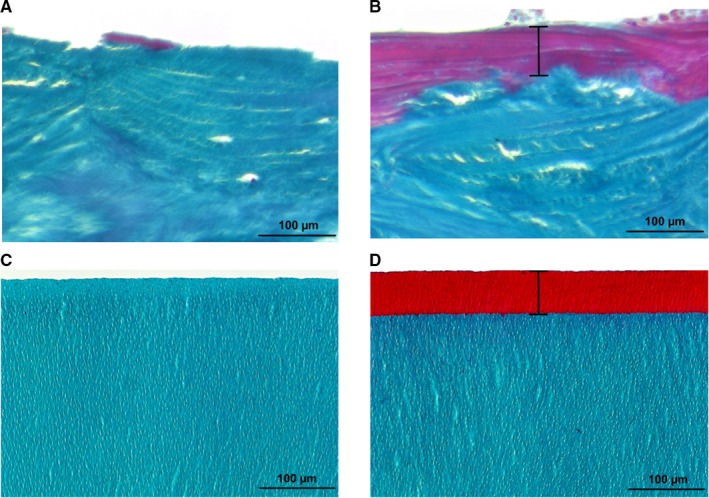
Comparison of osteoid seam and OIV model. Goldner trichrome stained bone biopsies of a healthy individual (A) and an osteomalacia patient (B). The osteoid seam (B) is presented in red and marked by a black bar. The same staining was performed with the mineralized surface (C) and OIV model (D) to visualize the degree of mineralization. (Light microscopy, magnification 20×)

While we could not detect an osteoid seam in the bone biopsy of a healthy patient (Figure 1A), histomorphometric examination of bone biopsies from patients with osteomalacia showed an osteoid seam of 57.8 ± 6.9 µm (Figure 1B). In our dentin model (Osteoidosis in vitro, OIV), we generated an unmineralized seam that was in the same order of magnitude (Figure 1D; 56.5 ± 1.0 µm). In the control specimens (Mineralized surface, MS), no zone of unmineralized matrix was detectable (Figure 1C).

### 3.2 Surface analysis

**FIGURE 2:**
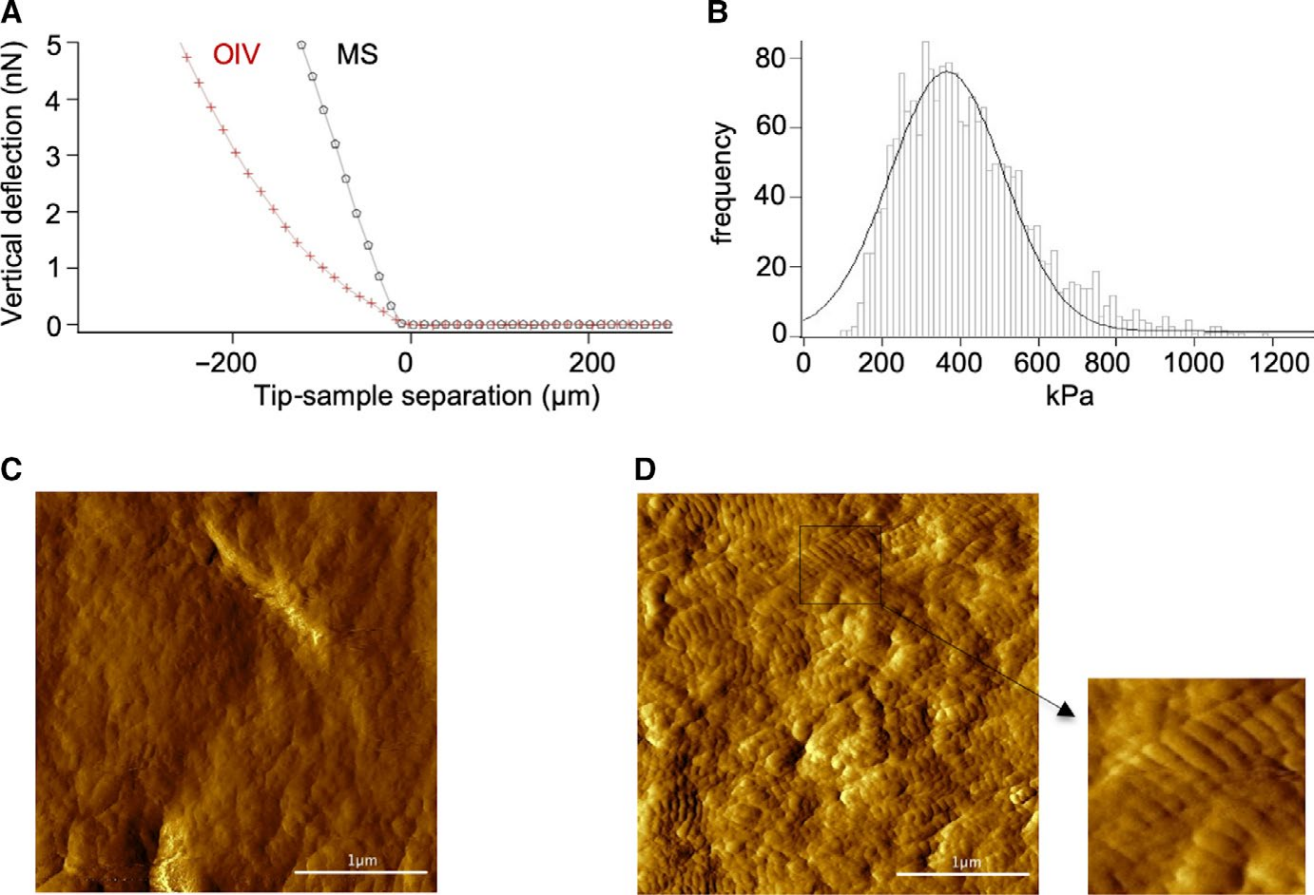
Surface mechanics. Force curves of MS and OIV (A) are shown. The histogram (B) depicts the distribution of the measured Young's modulus of OIV (curve corresponds to the Gaussian fit). Representative AFM images of MS (C) and OIV (D) are shown. The collagen network on OIV can be detected by the typical cross‐striation of the exposed collagen fibres

To quantify the changes in surface properties, we analysed MS and OIV by atomic force microscopy. The comparison of the force curves of MS and OIV indicated a substantial difference in the material's rigidity (Figure 2A) which is illustrated by the force increase which is much more pronounced on MS compared with OIV. Moreover, the Young's modulus of OIV was determined at 366 ± 4.3 kPa (Figure 2B) by atomic force microscopy (AFM). The Young's modulus of MS was so much higher than OIV that it exceeded the calibrated range of our equipment Other research groups have determined the Young's modulus of mineralized dentin at 18‐20 GPa.^27^, ^28^, ^29^ Thus, the unmineralized matrix in OIV leads to a decrease of tissue stiffness in the range of five powers of magnitude.

The comparison of AFM images further revealed the typical cross‐striation of exposed collagen fibrils on OIV (Figure 2D). The AFM image of MS, on the other hand, showed no cross‐striation (Figure 2C). The analysis of the D‐period (data not shown) confirmed the exposure of the collagen network due to demineralization. As expected, the distance between the collagen molecules was found to be 63.6 ± 3.6 nm.

### 3.3 Effects on the number of osteoclasts

**FIGURE 3:**
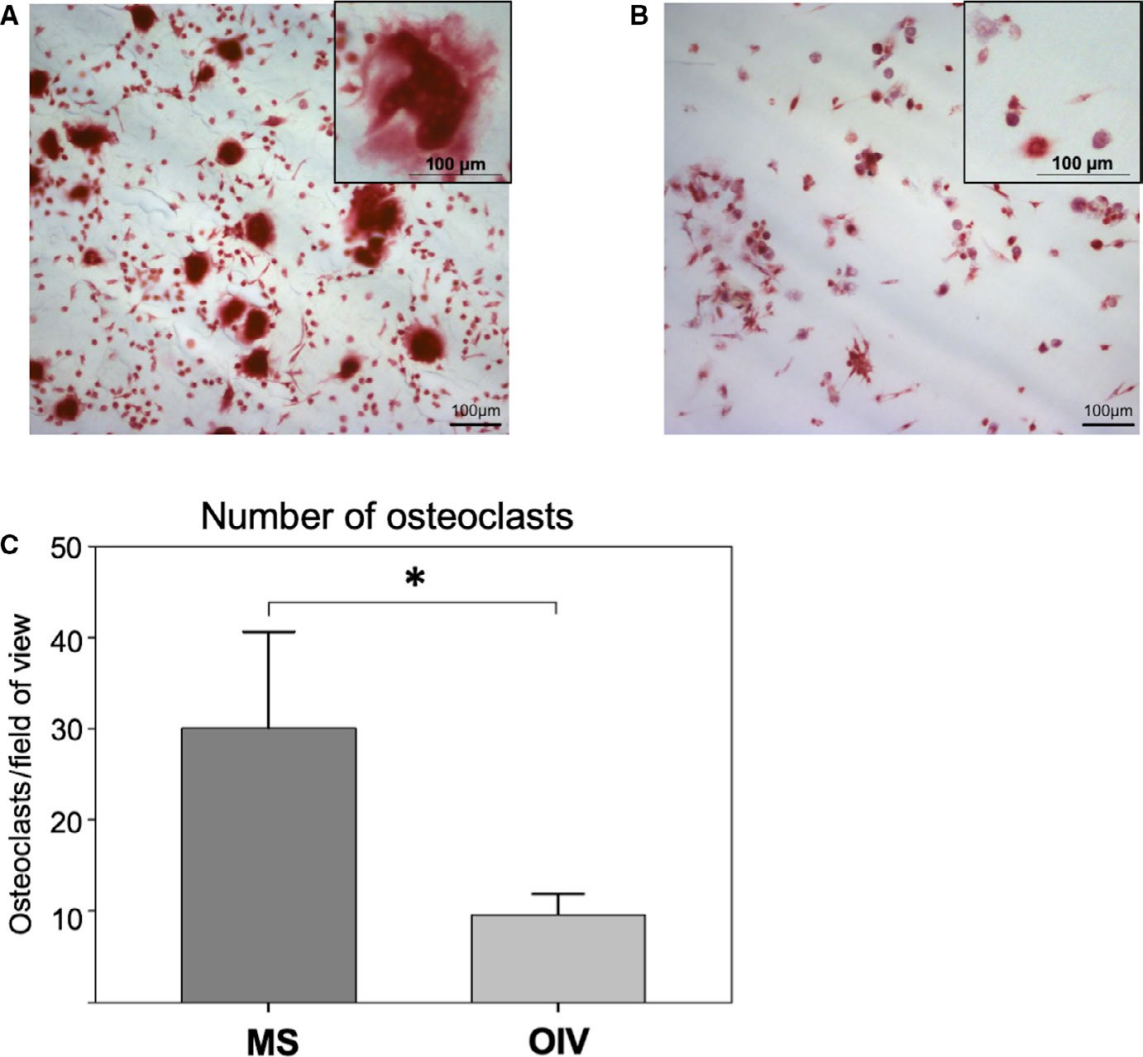
TRAP staining. TRAP^+^ multinucleated cells were detectable on MS (A, TRAP staining, magnification 10×), cells on OIV were also TRAP^+^ but mainly mononuclear (B). The number of osteoclasts showed a threefold reduction on OIV (C, *P < .05)

In comparison to MS (Figure 3A), there was a significantly lower number of differentiated osteoclasts after 28 days on OIV (Figure 3B); MS: 30.08 ± 8.60 osteoclasts/field of view vs OIV: 9.59 ± 1.88 osteoclasts/field of view, (Figure 3C, *P* = .03).

### 3.4 Quantification of resorption activity

**FIGURE 4:**
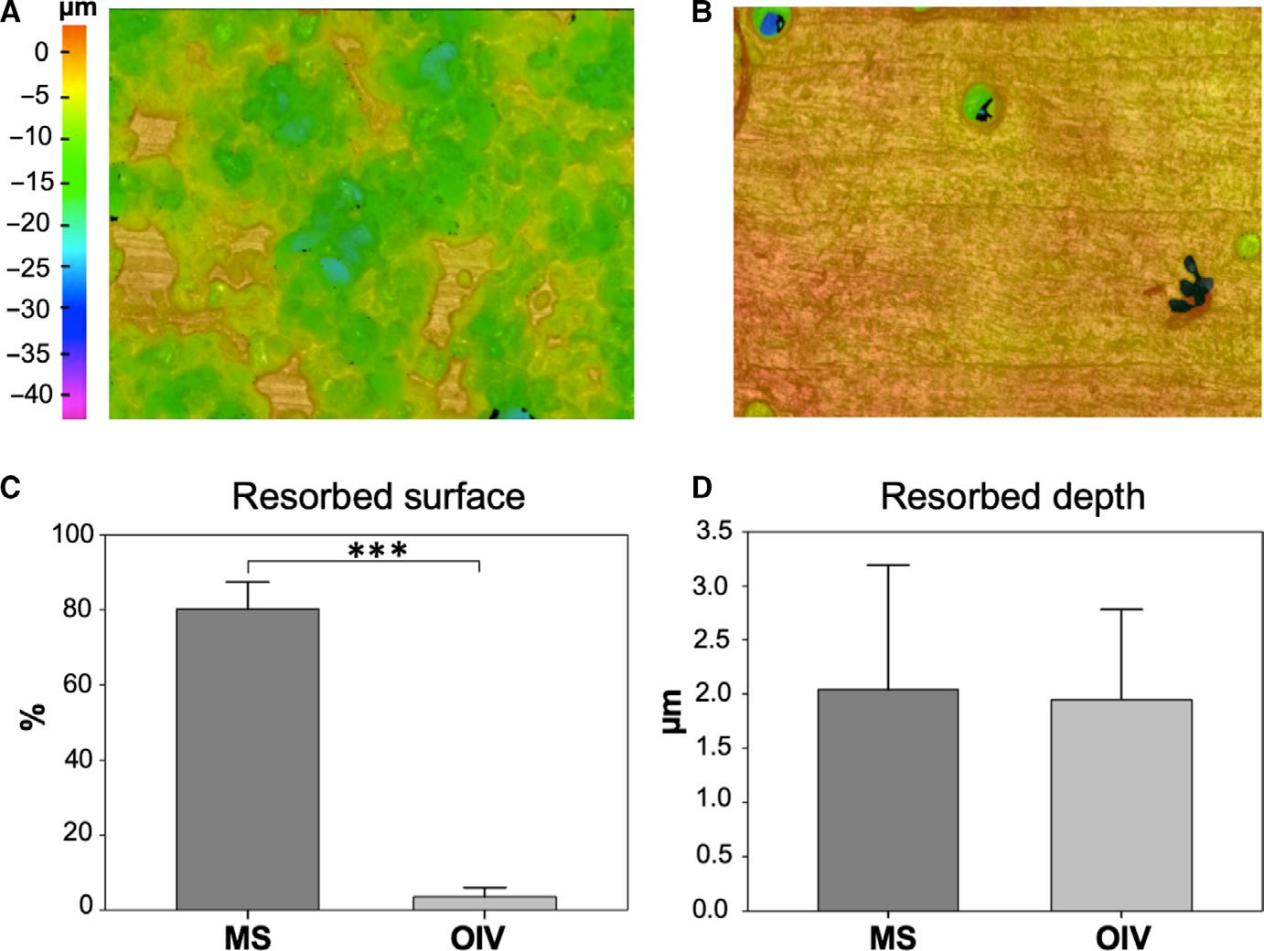
Resorption activity. Pseudocolour three‐dimensional IFM images of MS (A) and OIV (B) show the distribution and depth of resorption pits. The bar charts represent a significant difference in surface resorption (C), whereas the resorption depth (D) showed no difference (***P < .001)

Three‐dimensional quantification of osteoclastic resorption revealed a strong resorptive activity of osteoclasts on MS (Figure 4A,C, MS: 80.15 ± 7.22%) while there was almost no resorption detectable on OIV (Figure 4B,C, OIV: 3.54 ± 2.47%; *P* < .001). Interestingly, resorption depth of individual resorption pits was not significantly different between MS and OIV (Figure 4D, MS: 2.04 ± 1.15 µm vs OIV: 1.95 ± 0.83 µm; *P* = .896).

### 3.5 Resorption activity on RGD peptide coating

**FIGURE 5:**
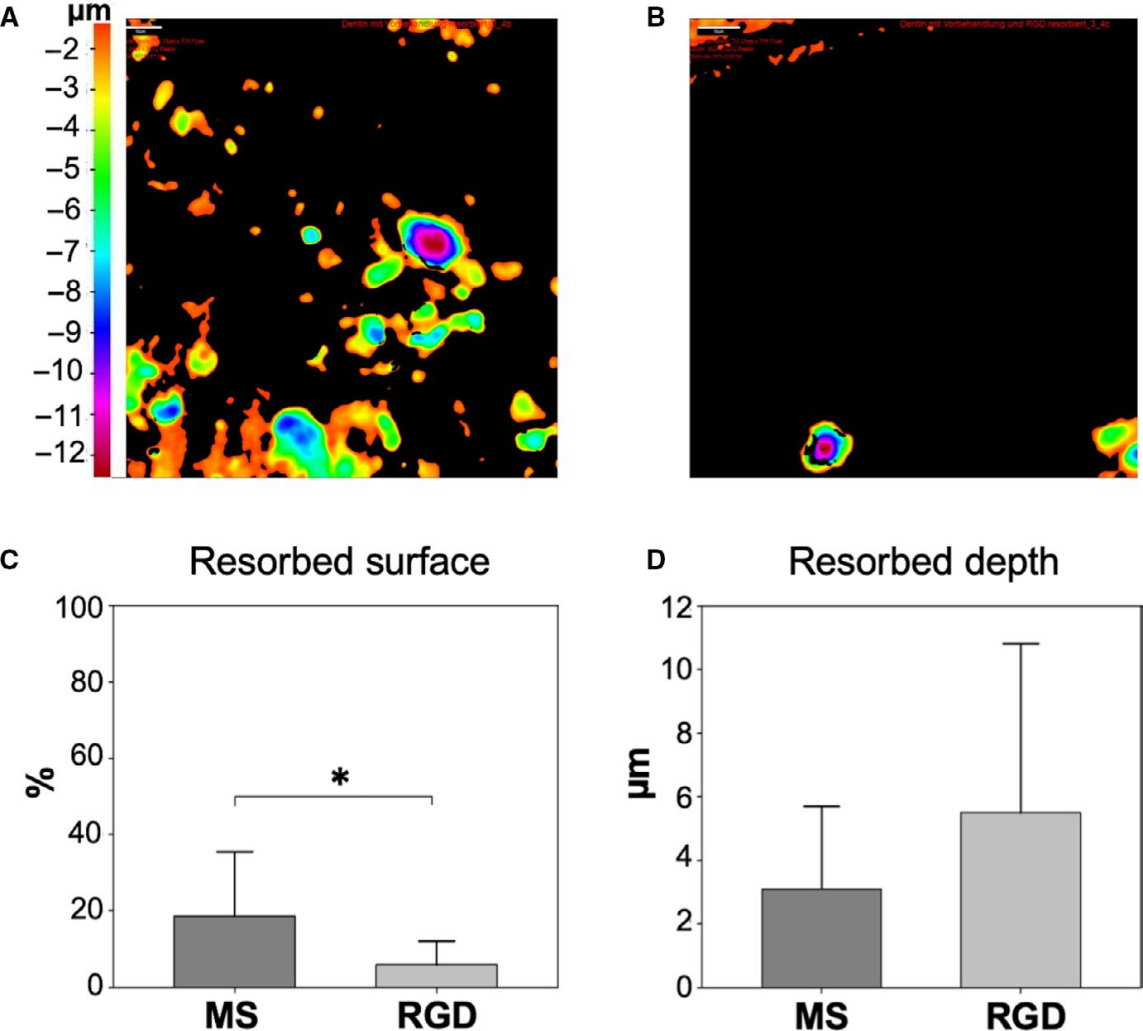
Resorption activity on RGD peptide coating. Pseudocolour three‐dimensional IFM images of MS (A) and RGD‐coated dentin (B) show the distribution and depth of resorption pits. The comparison of the surface resorption (C) revealed a statistically significant difference between MS and RGD, whereas the resorption depth (D) showed no difference (*P < .05)

As collagen is known to directly influence cell behaviour through its high level on RGD peptide, we coated MS with a very thin layer of RGD peptides, without detectable changes of the surface mechanics. Again, we found a strong reduction of resorption area on RGD‐coated dentin (Figure 5C, MS = 18.5 ± 17.0% vs RGD = 5.9 ± 6.2%; *P* = .01), while the resorption depth on MS and RGD did not differ significantly (Figure 5D, MS = 3.1 ± 2.6 µm vs RGD = 5.5 ± 5.3 µm; *P* = .15).

### 3.6 Analysis of gene expression

**FIGURE 6:**
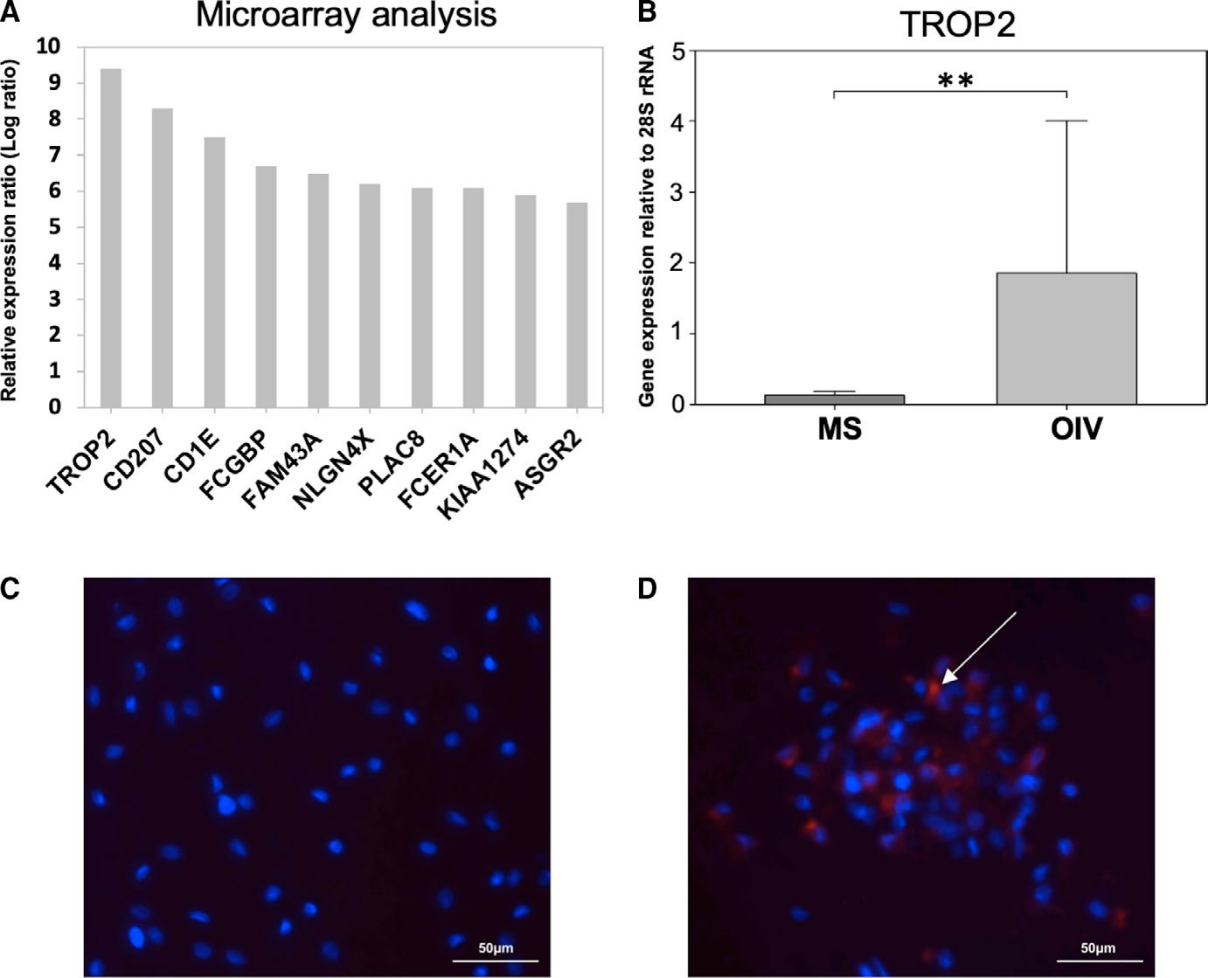
Expression of TROP2. mRNA microarray analysis shows the ten most up‐regulated genes on OIV (A). The bar chart (B) displays the increased expression of TROP2 on OIV compared with MS (*P < .05). Immunohistochemistry (fluorescence microscopy, magnification 40×) of MS (C) and OIV (D) shows TROP2^+^ cells (red, white arrow) on OIV. Dapi counterstaining (blue) was performed to visualize cell nuclei

To find an explanation for the significantly lower number of osteoclasts as well as the decreased resorption area on OIV, whole genome microarray analysis was performed for both groups after 28 days of cultivation (Figure 6A). We identified the tumour‐associated calcium signal transducer 2 (TROP2) to be the most strongly up‐regulated gene on OIV compared with MS which was confirmed by qPCR analysis (Figure 6B). On OIV, TROP2 showed a significant 15‐fold up‐regulation (Figure 6B, MS = 0.12 ± 0.06 vs OIV = 1.85 ± 2.15, *P* = .008).

### 3.7 TROP2^+^ cells on osteoidosis in vitro

TROP2 staining of cells on MS and OIV confirmed our qPCR results on a protein level. While we could not detect TROP2 in osteoclast cultures on MS (Figure 6C), there was a strong expression in the cells on OIV (Figure 6D).

### 3.8 TROP2^+^ osteoclasts in human osteomalacia

**FIGURE 7:**
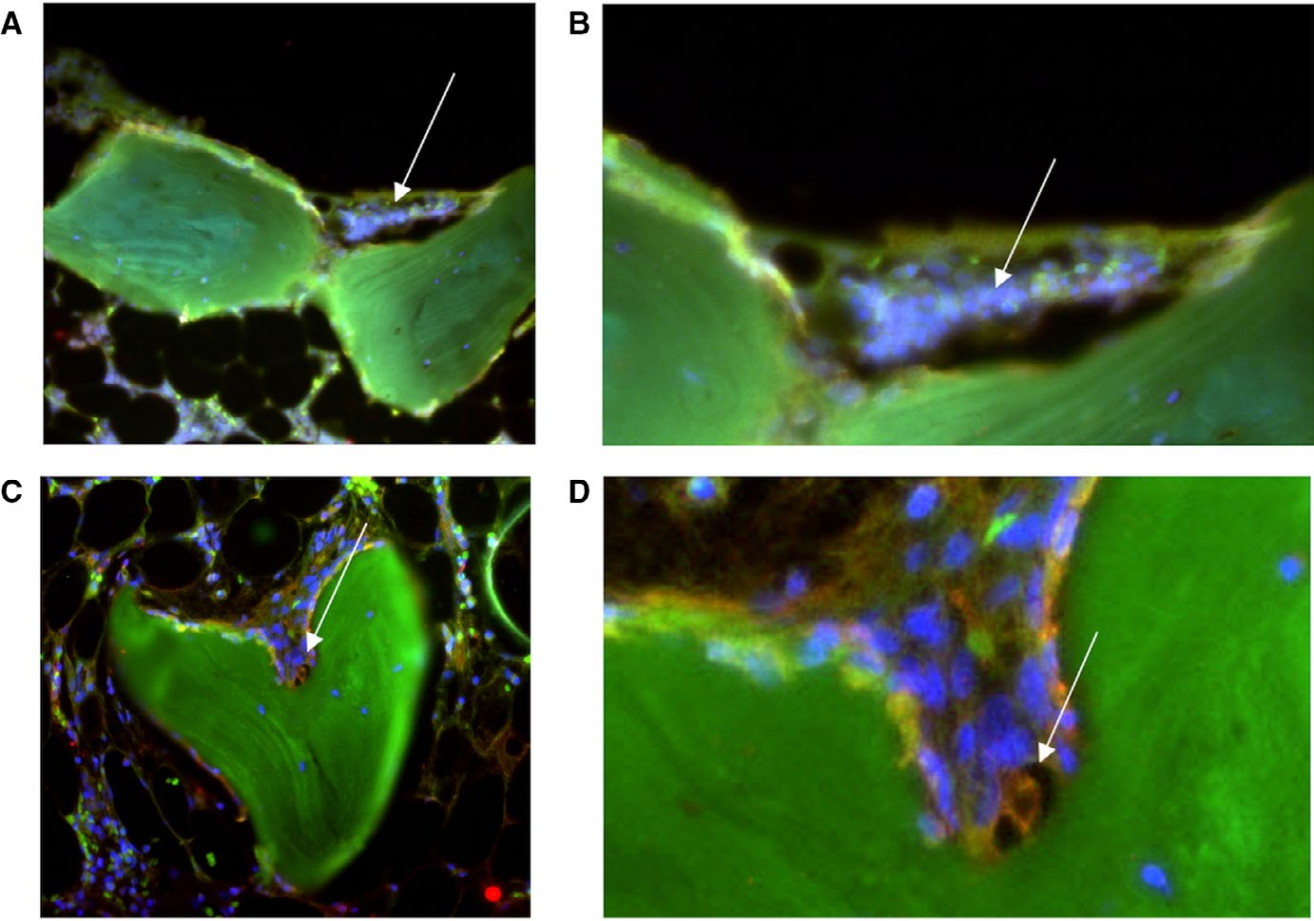
TROP2 in osteomalacia bone biopsy. TROP2‐positive osteoclasts (red) were also identified in an osteomalacia bone biopsy (C, fluorescence microscopy, magnification 20×) surrounding a resorption lacuna (green = autofluorescence of collagen). Dapi counterstaining (blue) was performed to visualize cell nuclei. (D) Enlarged image section. The control histology of healthy bone (A, B) showed no TROP2‐positive osteoclasts

Given the presence of osteoid on the bone surface in healthy individuals as well as in patients with osteomalacia, the increased thickness and pathologic accumulation of osteoid in relation to the remaining bone volume are pathognomonic for osteomalacia. The staining revealed no expression of TROP2 on osteoclasts on the bone surface of a healthy individual (Figure 7A,B). However, a strong expression of TROP2 could be detected on cells on the surface of unmineralized bone in a patient with osteomalacia (Figure 7C,D), while most cells in the surrounding bone marrow were not positive for TROP2. This suggests bone‐specific TROP2 expression in the context of osteomalacia.

## 4 DISCUSSION

We show here that a change of matrix mineralization, which can be found in several metabolic bone diseases, alters the function and differentiation of osteoclasts in vitro. This is most likely due to an exposition of collagen fibrils resulting in an RGD‐mediated pathway.

In our model system, we were able to generate a unmineralized seam that was of the same order of magnitude as that in bone biopsies from osteomalacia patients and consistent with Parfitt's definition of osteomalacia referring to a mean osteoid thickness above an absolute value of 12.5 µm.^11^

Although the same cell metabolic activity indicating a similar number of progenitor cells on OIV and MS was found at day 1 (MTT‐assay, Figure S1), the differentiation of human osteoclasts on OIV resulted in a significantly lower number of cells and a reduction of the resorbed surface.

Of note, the whole genome microarray revealed a 15‐fold up‐regulation of TROP2 expression on OIV while expression of osteoclastic differentiation markers was comparable for both groups (Figure S2). TROP 2 expression on OIV was further confirmed on a protein level with immunohistochemistry in vitro and in patients with osteomalacia. TROP2 is a cell surface receptor that is highly expressed in trophoblast cells, prostate basal cells and hepatic oval cells with stem cell characteristics.^30^, ^31^ Additionally, TROP2 has been found on TGF‐ß1‐dependent Langerhans cells,^32^ its functional role in these cells remains however elusive. Interestingly, all the localizations of reported TROP2 expression (placenta, skin, epithelial carcinomas, fibrotic liver) have a close relation to collagen‐rich ECM. It has so far not been detected in bone marrow or peripheral blood.^32^ Yang et al^33^ have shown that TROP2 is involved in the regulation of proliferation and differentiation of mesenchymal stem cells (MSCs). According to their data, TROP2 deficiency inhibited proliferation of MSCs and impaired differentiation by reducing adipogenesis and osteogenesis. Hence, they concluded that TROP2 expression is a crucial factor during osteogenesis and MSC self‐renewal in particular. In the specific context of pathologic decrease of bone matrix mineralization which can be found in various metabolic bone diseases such as osteomalacia, chronic kidney disease‐mineral bone disorder or hypophosphatasia, increased expression of TROP2 might act as a stimulator of MSC osteogenic differentiation in an attempt to improve the bone structure by osteoblasts.^34^, ^35^

The RGD sequence of the exposed collagen fibrils that were identified by their typical cross‐striation^36^ may directly be responsible for the altered differentiation of osteoclasts. RGD is a tripeptide amino acid sequence that occurs in proteins of the extracellular matrix and is known to play a key role in integrin‐mediated cell‐matrix adhesion.^37^ Based on further experiments, the effect of OIV could be mimicked by coating MS with RGD peptides. Similarly to OIV, the resorption area on RGD was reduced while the depth of the single resorption pit showed no difference. Given the significantly lower number on osteoclasts on OIV, we suggest an interaction between the osteoclastic vitronectin receptor and the RGD peptide that could lead to an inhibition of osteoclastic differentiation and up‐regulation of TROP2 expression. A further explanation for the observed effects might be a stronger adhesion of osteoclast precursors to the RGD sequence resulting in a decreased migration potential and impaired cytoskeletal organization which is necessary for resorption. These findings would be consistent with Shafieyan et al^38^ who differentiated osteoclasts on polydimethylsiloxane coated with collagen at different concentrations and found an inhibition of monocyte fusion by high collagen densities.

While the inhibitory effect of RGD‐containing proteins such as echistatin has been observed before, it was suggested that the occupation of the vitronection receptor prevented any attachment of osteoclasts to the RGD peptide of the extracellular matrix and thus cell spreading and resorption.^39^, ^40^, ^41^ In our experiments, the collagen‐containing RGD sequences were strongly attached to the surface, suggesting a direct effect on the precursor cells which challenges the original concept of competitive inhibition.

Another possible influence on cell differentiation could be mediated by surface mechanics. The strongly decreased Young's modulus of OIV may therefore be in part responsible for the expression of a gene that is typically found in much softer tissues. However, when we analysed the expression of typical mechanotransduction genes in our samples, we could find no significant changes between MS and OIV (Figure S3), making this hypothesis unlikely.

A limitation of our model system is that we used dentin instead of bone. However, despite their different composition and structure, we have shown previously that dentin and human bone are indistinguishable in terms of their osteoclastic resorption activity in vitro.^42^ Furthermore, our osteoidosis model is based on demineralization of dentin, whereas osteomalacia in human bone results from an impaired bone metabolism and lack of mineralization. Moreover, the extent of osteomalacia is variable among patients which might have different effects on the cells and should be subject of further research. Our model system is also limited by the fact that it leaves a number of physiological processes and especially the interaction between osteoclasts and osteoblasts unaddressed.

In conclusion, we show that a change of matrix mineralization alters the differentiation and function of human osteoclasts in vitro. The reduced number of osteoclasts and resorbed surface area was associated with an up‐regulation of TROP2 expression on an osteoidosis model as well as in patients with osteomalacia, which has not been described so far. This is possibly due to binding of precursor cells to unmasked extracellular matrix proteins containing RGD sequences, resulting in an RGD peptide‐mediated pathway. Based on our results, we suppose that the different surface characteristics of the extracellular matrix may be important for the pathophysiology of several metabolic bone diseases with a defect in matrix mineralization such as osteomalacia, rickets, chronic kidney disease‐mineral bone disorder or hypophosphatasia.

## CONFLICT OF INTEREST

The authors confirm that there are no conflicts of interest.

## AUTHOR CONTRIBUTIONS

LG, CP, TW, MK, UH, HCS and AFS designed the study and performed the research. LG, CP, TW and AFS analysed the data and wrote the paper. KK, TW, TS, JZ, MMM and HGM contributed to the establishment of the osteoidosis model. All authors approved the submitted and final version of the paper.

## Supporting information

Fig S1

Fig S2

Fig S3

## Data Availability

The data that support the findings of this study are available from the corresponding author upon reasonable request.
