# Supplementary figures and images for: Osteoidosis leads to altered differentiation and function of osteoclasts
Source: J Cell Mol Med. 2020 Apr 13;24(10):5665–74. doi: 10.1111/jcmm.15227 (PMC7214153; doi:10.1111/jcmm.15227)

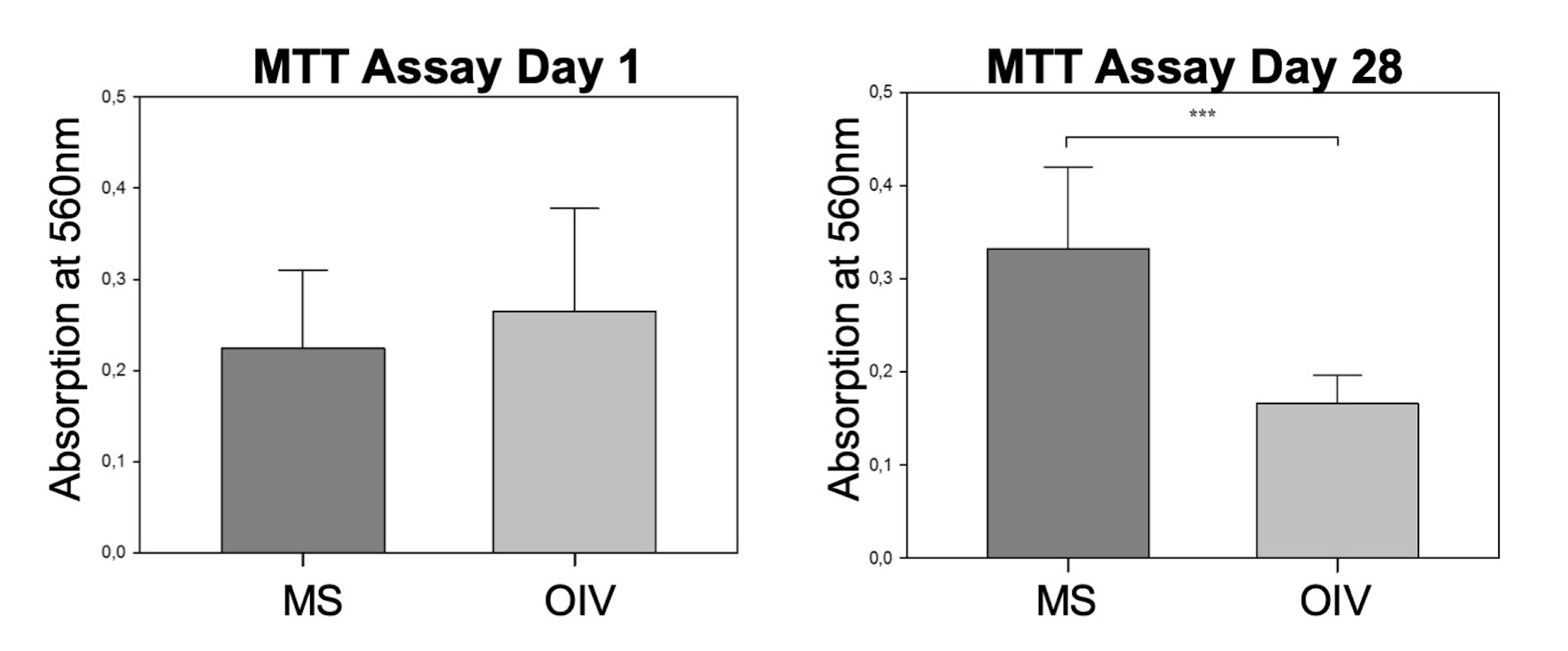

Supplement: Supplementary file 1 — Fig S1 [file JCMM-24-5665-s001.tif]

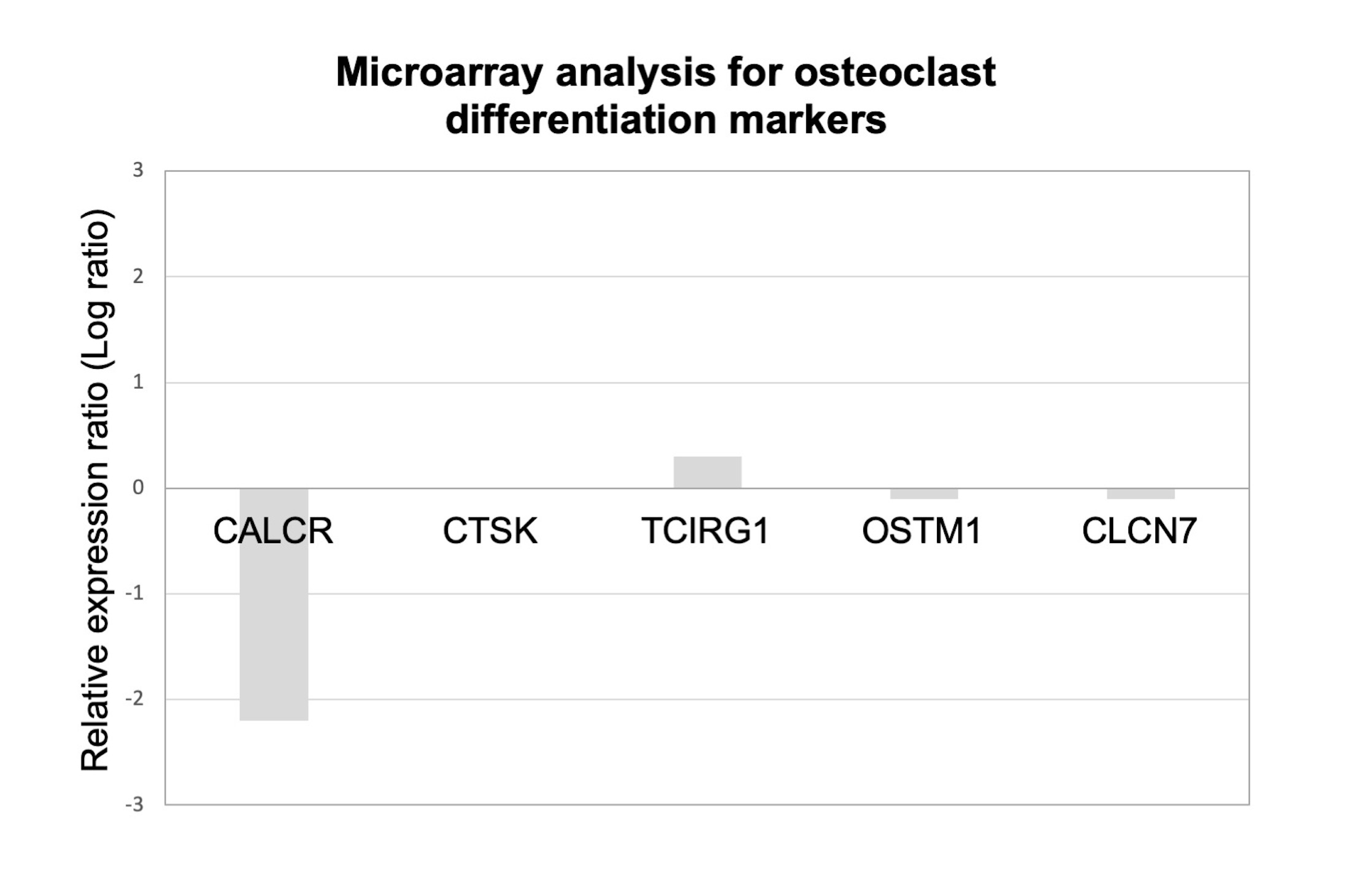

Supplement: Supplementary file 2 — Fig S2 [file JCMM-24-5665-s002.tif]

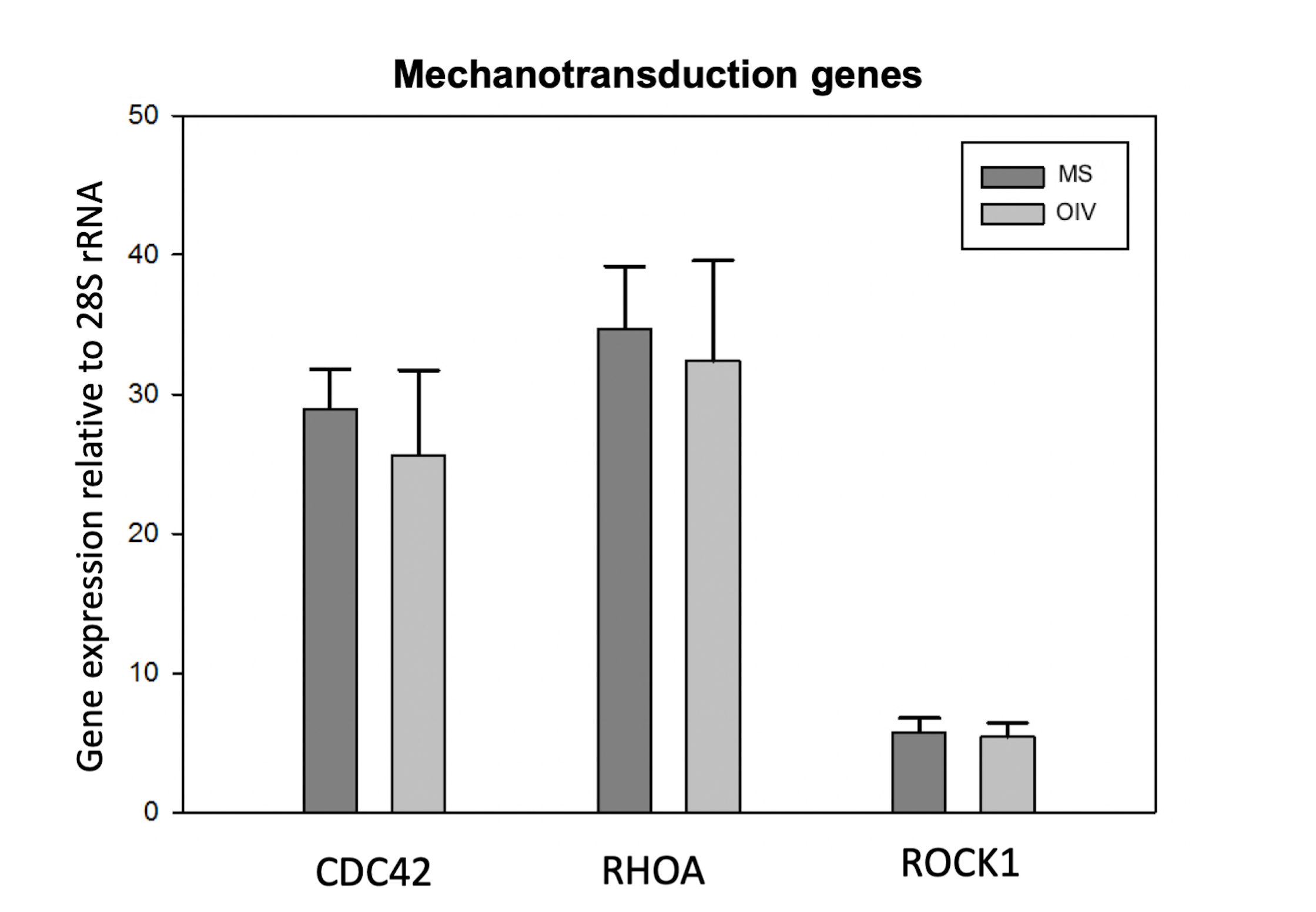

Supplement: Supplementary file 3 — Fig S3 [file JCMM-24-5665-s003.tif]
